# Decoding the mechanisms of acupuncture by neuroimaging: an integrated review from networks to molecules

**DOI:** 10.3389/fnhum.2026.1704570

**Published:** 2026-01-30

**Authors:** Shiping Liu, Yan Bai, Jie Liu, Xia Chen, Peizhu Lv, Yulin Wang, Dandan Wang, Shun Wang

**Affiliations:** 1 Heilongjiang University of Traditional Chinese Medicine, Harbin, China; 2 Heilongjiang Provincial Academy of Traditional Chinese Medicine Sciences, Harbin, China; 3 The Second Affiliated Hospital of Heilongjiang University of Chinese Medicine, Harbin, China

**Keywords:** acupuncture, brain, EEG, fMRI, molecular mechanisms, multimodal integration, networks, neuroimaging

## Abstract

Acupuncture, a cornerstone of Traditional Chinese Medicine (TCM), is widely used for conditions like chronic pain and functional disorders, yet its neurobiological mechanisms are not fully understood. This review synthesizes findings from multimodal neuroimaging–including functional magnetic resonance imaging (fMRI), electroencephalography (EEG), and positron emission tomography (PET)–to examine the central nervous system and neurochemical correlates of acupuncture. We summarize reports of its modulatory effects on large-scale brain networks (e.g., Default Mode Network, Salience Network) and neural oscillations, alongside evidence of neurochemical changes. Importantly, we also address the methodological limitations, inconsistent results, and significant role of non-specific (e.g., placebo) effects prevalent in this literature (Chen B. et al., 2023; Yu et al., 2024). Building on this evidence, we propose a multi-level integrative framework that outlines a potential pathway from peripheral stimulation to clinical outcomes via neurochemical and network-level interactions, while carefully distinguishing observed correlations from established causation. We conclude by discussing future research priorities, emphasizing the need for standardized protocols, rigorous causal inference, and a measured approach to translating emerging technologies. This review aims to bridge traditional practice with modern neuroscience by offering a balanced perspective that highlights both progress and persistent challenges in the field.

## 1 Introduction

### 1.1 Clinical efficacy and the mechanistic challenge

With a history spanning millennia, acupuncture is globally utilized for conditions such as chronic low back pain (Wen et al., 2021), various pain syndromes (Pock and Niemtzow, 2021), Alzheimer’s disease (Cai and Yang, 2020), and functional dyspepsia (Dong et al., 2022). Despite its clinical application, the biological mechanisms underlying its effects remain incompletely defined. While traditional concepts like “Qi” and “meridians” offer one explanatory framework, a gap persists with modern biomedical understanding (Hui et al., 2000). Closing this gap through robust mechanistic evidence is essential for acupuncture’s integration into contemporary evidence-based medicine.

### 1.2 Neuroimaging: a window into central correlates

Non-invasive neuroimaging has opened a valuable window into the brain’s response to acupuncture (Napadow et al., 2009). Early research focused on identifying activated or deactivated brain regions (Fang et al., 2009). The field has since progressed toward a systems-level approach, investigating how acupuncture might influence the dynamics of large-scale brain networks and neural circuits (Dhond et al., 2007; Napadow et al., 2013). Yet, interpreting these neuroimaging findings demands careful acknowledgment of their inherent correlational nature and the various confounds present in acupuncture studies.

### 1.3 Review scope and critical perspective

This review provides a comprehensive and critical overview of current neuroimaging evidence concerning acupuncture mechanisms. We will: (1) Detail the insights and constraints of established techniques (fMRI, EEG) in revealing brain network and neural dynamics; (2) Discuss the emerging, though often preliminary, role of molecular imaging (PET) and novel technologies in probing neurochemical substrates; (3) Integrate these findings into a coherent, multi-level framework, while explicitly addressing the predominance of correlational data and the challenges in establishing causality; and (4) Identify key methodological issues, contradictory findings, and outline future directions that prioritize scientific rigor.

## 2 Established neuroimaging techniques: mapping macroscopic brain dynamics

### 2.1 Functional magnetic resonance imaging (fMRI)

Functional magnetic resonance imaging, particularly using the blood oxygenation level-dependent (BOLD) signal, has been extensively employed to map brain responses to acupuncture with high spatial resolution.

Studies suggest that needling at specific acupoints, such as ST36 (Zusanli) and LI4 (Hegu), can modulate activity within a distributed network involving the limbic system (e.g., amygdala, hippocampus), paralimbic regions (e.g., anterior cingulate cortex, insula), and subcortical structures (e.g., hypothalamus) (Chae et al., 2013; Hui et al., 2000). This network is integral to pain perception, emotion, and autonomic control, potentially underpinning acupuncture’s diverse effects.

Resting-state fMRI (rs-fMRI) research indicates that acupuncture may alter functional connectivity within and between large-scale intrinsic brain networks. A commonly observed finding is a decrease in Default Mode Network (DMN) connectivity following acupuncture (Fan et al., 2020; Wang et al., 2017). Since the DMN is active during self-referential thought, its deactivation aligns with subjective reports of relaxation post-treatment (Dhond et al., 2008). Acupuncture has also been shown to modulate the functional connectivity of the Salience Network (SN). For instance, in patients with low back pain, the analgesic effect of real acupuncture (with needle sensation) was specifically associated with decreased connectivity between the posterior insula (a key node of the SN) and the default mode network, highlighting the role of SN in processing salient somatosensory stimuli like Deqi (Lee et al., 2019). And may influence the Central Autonomic Network (CAN), relevant to visceral regulation (Li et al., 2024; Sun et al., 2021).

These fMRI findings must be considered alongside significant methodological constraints and inconsistent results. The BOLD signal is an indirect measure of neural activity. More critically, the specificity of acupuncture’s effects on brain networks is debated. Reviews indicate that while some studies report distinct patterns for real versus sham acupuncture, others demonstrate considerable overlap, with sham procedures often eliciting similar, if somewhat attenuated, brain modulation (Chen B. et al., 2023; Kelly et al., 2024). This highlights the substantial impact of non-specific factors like expectation and general somatosensory stimulation. Additional challenges include motion artifacts during needling, frequently small sample sizes, a historical lack of study pre-registration, and the inherent difficulty in designing truly inert control interventions.

### 2.2 Electroencephalography (EEG)

Electroencephalography measures neuronal electrical activity directly with millisecond resolution, capturing rapid dynamics.

Acupuncture has been associated with changes in oscillatory power across frequency bands. An increase in alpha power (8–13 Hz), particularly over parietal and occipital areas, is a frequently reported finding linked to states of relaxed wakefulness (Yu et al., 2024). Changes in beta (13–30 Hz) and gamma (>30 Hz) activity have also been noted, possibly related to somatosensory processing and attention (Nierhaus et al., 2015). The temporal precision of EEG allows analysis of event-related potentials (ERPs) synchronized to needle manipulation.

Variability and Interpretation: Considerable variability exists across EEG studies in the topography, direction, and consistency of these oscillatory changes. Outcomes are influenced by needling parameters, subject state, and control conditions. This heterogeneity means EEG responses to acupuncture are not uniform. As with fMRI, a central challenge remains distinguishing effects specific to acupuncture from generalized responses to sensory stimulation and attentional shifts.

### 2.3 Complementary use and shared constraints of fMRI and EEG

Combining fMRI and EEG can powerfully correlate spatial networks with temporal dynamics. Concurrent EEG-fMRI studies may link fast oscillations with slower hemodynamic changes (Liang et al., 2024; Zhang et al., 2025). However, this multimodal approach also inherits and combines the limitations of each technique, adding complexity to experimental design and data interpretation.

## 3 Emerging molecular and functional imaging approaches

While fMRI and EEG map macroscopic correlates, they lack biochemical specificity. Newer molecular and functional imaging technologies promise greater specificity but remain at early stages of application in acupuncture research.

### 3.1 Positron emission tomography (PET)

Positron emission tomography enables quantitative measurement of specific neurochemical processes via radiotracers. Though limited in number, PET studies offer more direct biochemical insights.

A key application is investigating neurotransmitter systems. For example, using the radioligand [^11^C]carfentanil, PET has shown that verum acupuncture can alter μ-opioid receptor availability in certain brain regions compared to sham, supporting a role for the opioid system in some contexts of acupuncture analgesia (Harris et al., 2009). PET with ^1^8F-fluorodeoxyglucose (^1^8F-FDG), a measure of cerebral glucose metabolism, has suggested that electroacupuncture can modulate metabolism in brain regions relevant to conditions like Alzheimer’s disease in animal and human studies (Xu et al., 2020a,b).

Considerations: PET studies in acupuncture are few and resource-intensive. Their findings, while valuable, are not yet consistent across all populations or paradigms. They demonstrate the potential for neurochemical modulation but do not establish a definitive neurochemical signature for acupuncture.

### 3.2 Novel technologies and integrative approaches

Several emerging technologies offer new avenues for investigation, though their contributions are currently preliminary.

Functional near-infrared spectroscopy (fNIRS): This portable tool monitors cortical hemodynamics in clinical settings. Studies have used fNIRS to observe altered prefrontal activation during scalp acupuncture in stroke patients (Lin et al., 2025; Zhong et al., 2025). Hyperscanning studies also report increased inter-brain synchrony between practitioner and patient during needling (Chen L. et al., 2023), hinting at a neural basis for the therapeutic interaction. However, it is important to note that fNIRS is limited by its shallow penetration depth and remains susceptible to systemic physiological confounds unrelated to neural activity.

Bio-inspired and nanobiosensors: Integrating micro/nanotechnology with acupuncture needles has produced innovative biosensors. For instance, a transistor-based neuroprobe modeled on an acupuncture needle enabled real-time neurotransmitter monitoring *in vivo* (Zhou et al., 2022). Similarly, microneedle biosensors have monitored molecular release (e.g., hydrogen sulfide) in animal models after electroacupuncture (Pei et al., 2025). While these engineered devices represent a transformative toolset for basic science with high potential, a critical perspective is necessary: their use in acupuncture research is currently almost entirely confined to preclinical animal models. Translation to human studies faces major challenges regarding safety, biocompatibility, and practicality. Therefore, data from these platforms should be viewed as hypothesis-generating for human mechanisms rather than as direct evidence. They primarily illustrate technological possibilities rather than confirm established human pathophysiology.

Multimodal integration and artificial intelligence: Combining data from multiple imaging modalities and applying AI (e.g., machine learning) is a growing trend to overcome single-method limitations. AI may help identify complex patterns, predict treatment response from baseline scans, or assist with data processing (Ma et al., 2024; Zhang et al., 2025). Methodological frameworks like “mFusion” have been proposed to bridge imaging and genetic data via neurotransmitter systems (Cao et al., 2024). However, a note of caution is warranted: AI applications in acupuncture neuroimaging are still nascent. Risks include overfitting models to small, heterogeneous datasets and the limited interpretability of derived features, the so-called “black box” problem. At present, AI’s primary value lies in exploratory analysis and generating new, testable hypotheses rather than in providing definitive biomarkers.

## 4 Toward an integrative multi-level framework

A central aim of this field is to integrate multimodal findings into a coherent explanatory framework while progressively testing causal hypotheses.

### 4.1 Integrating multimodal evidence critically

Evidence convergence across modalities can strengthen inferences but does not equal proof of cause. Truly multimodal studies–like combined PET-fMRI to link neurochemical release with network changes, or concurrent EEG-fMRI to trace temporal sequences–are rare but crucial for advancing beyond correlation.

### 4.2 A proposed multi-level framework

**FIGURE 1:**
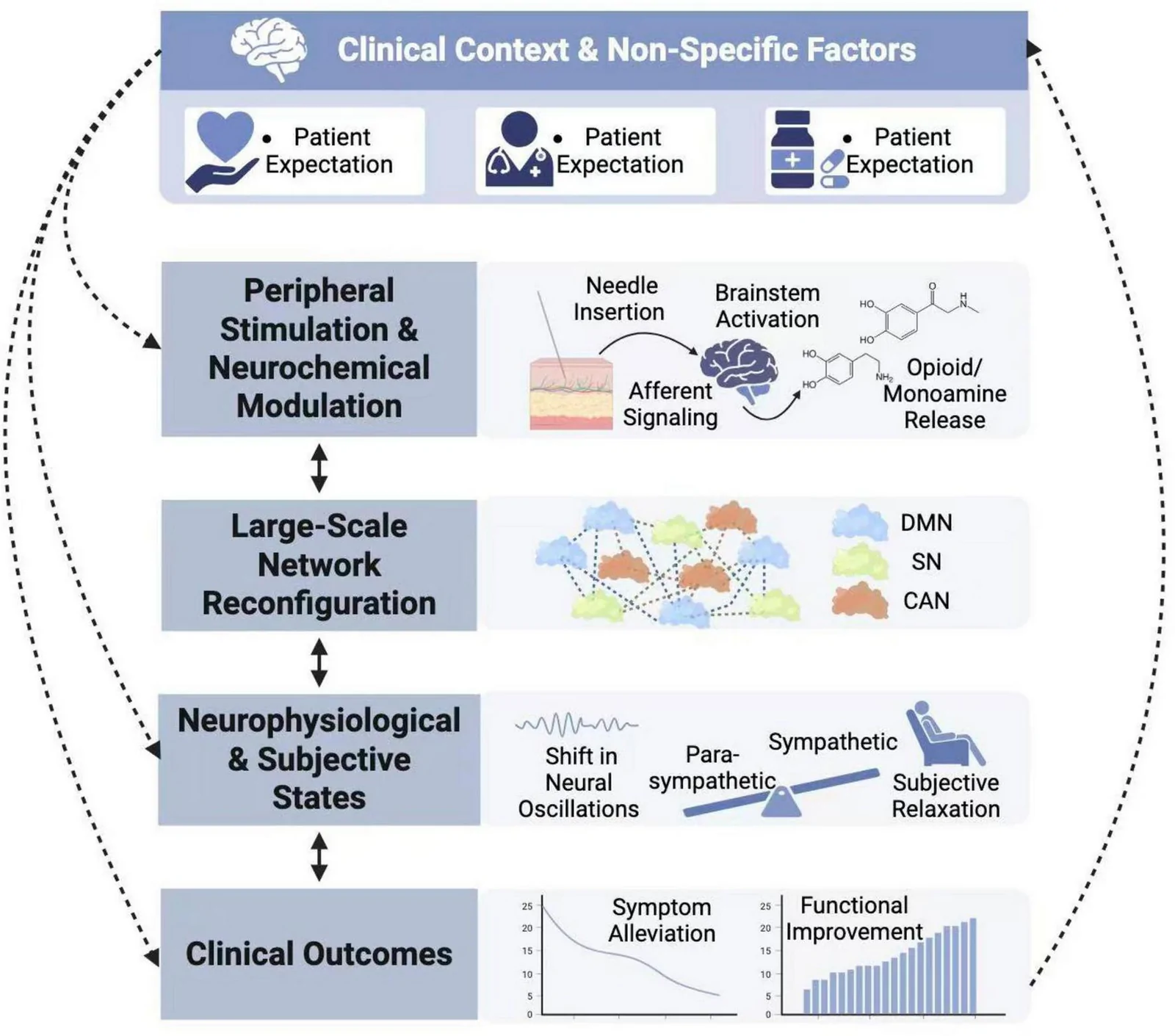
A proposed multi-level framework of acupuncture mechanisms (arrows indicate hypothesized relationships based on current evidence; establishing definitive causal links is a primary objective for future research).

Synthesizing the correlative evidence, we propose a multi-level framework that outlines a hypothesized pathway from peripheral stimulation to clinical outcome (Figure 1). This framework organizes existing findings into a plausible sequence while noting that causal connections require further validation.

The proposed sequence starts with peripheral needle insertion, evoking local biochemical and neural responses (Wu et al., 2024; Zhang et al., 2008). Signals are relayed to brainstem regions like the periaqueductal gray (PAG) (Napadow et al., 2009). Evidence from ligand studies suggests this may trigger neurochemical modulation, including release of endogenous opioids and monoamines (Cui et al., 1999; Harris et al., 2009). We hypothesize that these neurochemical shifts could contribute to a reconfiguration of large-scale brain networks. For instance, opioid release might be involved in altering connectivity within the descending pain pathway and affective networks (Wang et al., 2017; Yu et al., 2020). Such network-level changes, which include modulation of the DMN and SN, are correlated with subjective reports of altered self-awareness, attention, and autonomic state (Dhond et al., 2008; Lee et al., 2019; Sun et al., 2021; Yu et al., 2024). These network dynamics are reflected in shifts in neural oscillations, such as increased alpha power (Nierhaus et al., 2015; Yu et al., 2024). The ultimate clinical benefit likely arises from the combined influence of these processes.

It is crucial to maintain a key distinction here: this framework is built largely on correlations observed across different studies. Demonstrating that needle-induced neurochemical release directly causes a specific network change, which then directly causes a specific clinical improvement, remains an unmet goal. The model therefore serves as a working guide for designing future research that can test these proposed links, for example through pharmacological challenges combined with neuroimaging.

## 5 Future directions and ongoing challenges

### 5.1 Technical and analytical advances

Advances in imaging hardware (e.g., ultra-high-field MRI, next-generation PET) will enhance resolution and sensitivity. AI and computational models offer promising tools for analyzing complex datasets and predicting outcomes (Ma et al., 2024; Zhang et al., 2025). Their application, however, must be grounded in methodologically sound studies to avoid generating misleading results.

### 5.2 Methodological priorities

The credibility of future research hinges on addressing current shortcomings. A critical priority is enhancing rigor and standardization. The field urgently needs consensus on standardized protocols for acupoint location, needling technique, and–most critically–the selection and reporting of control conditions (sham/placebo). Pre-registration of trials and analytical plans is vital to improve reproducibility and reduce bias.

Simultaneously, future studies must proactively confront non-specific effects. Employing robust experimental designs, such as validated sham needles and effective blinding strategies, is essential to disentangle the specific physiological effects of needling from the powerful contextual, placebo, and somatosensory effects inherent in the therapeutic encounter.

Furthermore, research should systematically account for individual differences to pave the way for personalized approaches. This includes integrating factors like genetic polymorphisms [e.g., in the COMT gene, known to affect pain modulation (Ho et al., 2020)], psychological traits, and baseline brain architecture. Pretreatment neuroimaging has already shown preliminary value in predicting clinical response to acupuncture (Yu et al., 2020).

### 5.3 The path to translation

A major translational goal is the development of validated neuroimaging biomarkers capable of predicting treatment response or objectively verifying target engagement. Achieving this will require a concerted effort through large-scale, longitudinal, multicenter trials that incorporate neuroimaging as a core component. Fostering closer collaboration between neuroscientists, methodologies, and clinical acupuncturists is equally essential to ensure that research questions remain both biologically insightful and directly relevant to clinical practice.

## 6 Conclusion

Neuroimaging has significantly advanced the exploration of acupuncture’s mechanisms, shifting the discourse toward identifiable neurobiological correlates. Established techniques have mapped its influence on brain network dynamics and electrical activity, while molecular imaging has begun to illuminate potential neurochemical underpinnings. Integrating these strands of evidence allows for the construction of a multi-level framework that hypothesizes how needling might link to neurobiological events and clinical outcomes. Nevertheless, the current evidence landscape is characterized by correlation, heterogeneity, and notable methodological limitations. The clinical utility of acupuncture demands a research trajectory that places scientific rigor at its core. Moving forward, the field must leverage advanced technologies within robust experimental designs, rigorously address non-specific effects, and actively test the causal relationships within proposed mechanistic frameworks. By following this rigorous and transparent path, research can work toward a solidly evidence-based understanding of acupuncture’s place in integrative healthcare.

## References

[B1] Cai M. Yang E. (2020). Effect of combined electroacupuncture and selegiline treatment in Alzheimer’s disease: An animal model. *Front. Pharmacol.* 11:606480. 10.3389/fphar.2020.606480 33362561 PMC7758426

[B2] Cao L. Wang Z. Yuan Z. Luo Q. (2024). mFusion: A multiscale fusion method bridging neuroimages to genes through neurotransmissions in mental health disorders. *Commun. Biol.* 7:1699. 10.1038/s42003-024-07404-x 39719509 PMC11668864

[B3] Chae Y. Chang D. Lee S. Jung W. Lee I. Jackson S. (2013). Inserting needles into the body: A meta-analysis of brain activity associated with acupuncture needle stimulation. *J. Pain* 14 215–222. 10.1016/j.jpain.2012.11.011 23395475

[B4] Chen B. Guo Q. Zhang Q. Di Z. Zhang Q. (2023). Revealing the central mechanism of acupuncture for primary dysmenorrhea based on neuroimaging: A narrative review. *Pain Res. Manag.* 2023:8307249. 10.1155/2023/8307249 36852393 PMC9966569

[B5] Chen L. Qu Y. Cao J. Liu T. Gong Y. Tian Z. (2023). The increased inter-brain neural synchronization in prefrontal cortex between simulated patient and acupuncturist during acupuncture stimulation: Evidence from functional near-infrared spectroscopy hyperscanning. *Hum. Brain Mapp.* 44 980–988. 10.1002/hbm.26120 36255178 PMC9875919

[B6] Cui M. Feng Y. McAdoo D. Willis W. (1999). Periaqueductal gray stimulation-induced inhibition of nociceptive dorsal horn neurons in rats is associated with the release of norepinephrine, serotonin, and amino acids. *J. Pharmacol. Exp. Ther.* 289 868–876. 10.1016/S0022-3565(99)38213-8 10215665

[B7] Dhond R. Kettner N. Napadow V. (2007). Neuroimaging acupuncture effects in the human brain. *J. Altern. Complement. Med.* 13 603–616. 10.1089/acm.2007.7040 17718643

[B8] Dhond R. Yeh C. Park K. Kettner N. Napadow V. (2008). Acupuncture modulates resting state connectivity in default and sensorimotor brain networks. *Pain* 136 407–418. 10.1016/j.pain.2008.01.011 18337009 PMC2440647

[B9] Dong X. Yin T. Yu S. He Z. Chen Y. Ma P. (2022). Neural responses of acupuncture for treating functional dyspepsia: An fMRI study. *Front. Neurosci.* 16:819310. 10.3389/fnins.2022.819310 35585920 PMC9108289

[B10] Fan D. Zhao H. Sheng J. Liu Y. Yu J. (2020). Electroacupuncture modulates resting-state functional connectivity in the default mode network for healthy older adults. *J. Geriatr. Psychiatry Neurol.* 33 85–92. 10.1177/0891988719868304 31409183

[B11] Fang J. Jin Z. Wang Y. Li K. Kong J. Nixon E. (2009). The salient characteristics of the central effects of acupuncture needling: Limbic-paralimbic-neocortical network modulation. *Hum. Brain Mapp.* 30 1196–1206. 10.1002/hbm.20583 18571795 PMC6871074

[B12] Harris R. Zubieta J. Scott D. Napadow V. Gracely R. Clauw D. (2009). Traditional Chinese acupuncture and placebo (sham) acupuncture are differentiated by their effects on mu-opioid receptors (MORs). *Neuroimage* 47 1077–1085. 10.1016/j.neuroimage.2009.05.083 19501658 PMC2757074

[B13] Ho K. Wallace M. Staud R. Fillingim R. (2020). OPRM1, OPRK1, and COMT genetic polymorphisms associated with opioid effects on experimental pain: A randomized, double-blind, placebo-controlled study. *Pharmacogenomics J.* 20 471–481. 10.1038/s41397-019-0131-z 31806881 PMC7260086

[B14] Hui K. Liu J. Makris N. Gollub R. Chen A. Moore C. (2000). Acupuncture modulates the limbic system and subcortical gray structures of the human brain: Evidence from fMRI studies in normal subjects. *Hum. Brain Mapp.* 9 13–25. 10.1002/(sici)1097-019320009:1<13::aid-hbm2<3.0.co;2-f 10643726 PMC6871878

[B15] Kelly N. Mansfield C. Schneider E. Moeller J. Bleacher J. Prakash R. (2024). Functional connectivity patterns are altered by low back pain and cause different responses to sham and real dry needling therapies: A systematic review of fMRI studies. *Physiother. Theory Pract.* 40 671–688. 10.1080/09593985.2022.2155094 36484262

[B16] Lee J. Eun S. Kim J. Lee J. Park K. (2019). Differential influence of acupuncture somatosensory and cognitive/affective components on functional brain connectivity and pain reduction during low back pain state. *Front. Neurosci.* 13:1062. 10.3389/fnins.2019.01062 31636536 PMC6788296

[B17] Li J. Peng C. He K. Wang Y. Lai X. (2024). The central mechanisms of electroacupuncture at LR3 in the treatment of spontaneous hypertension: A PET and mRNA transcriptome study. *Front. Cardiovasc. Med.* 11:1358426. 10.3389/fcvm.2024.1358426 39234603 PMC11371727

[B18] Liang X. Guo Y. Zhang H. Wang X. Li D. Liu Y. (2024). Neuroimaging signatures and a deep learning modeling for early diagnosing and predicting non-pharmacological therapy success for subclinical depression comorbid sleep disorders in college students. *Int J Clin Health Psychol.* 24:100526. 10.1016/j.ijchp.2024.100526 39759571 PMC11699106

[B19] Lin B. Ni J. Xiong X. Zhang L. Song J. Wang M. (2025). Language function improvement and cortical activity alteration using scalp acupuncture coupled with speech-language training in post-stroke aphasia: A randomised controlled study. *Complement. Ther. Med.* 89:103137. 10.1016/j.ctim.2025.103137 39892714

[B20] Ma L. Chen S. Zhang Y. Qin X. Wu X. (2024). Integration patterns of functional brain networks can predict the response to abdominal acupuncture in patients with major depressive disorder. *Neuroscience* 560 286–296. 10.1016/j.neuroscience.2024.10.002 39368604

[B21] Napadow V. Dhond R. Park K. Kim J. Makris N. Kwong K. (2009). Time-variant fMRI activity in the brainstem and higher structures in response to acupuncture. *Neuroimage* 47 289–301. 10.1016/j.neuroimage.2009.03.060 19345268 PMC2692758

[B22] Napadow V. Lee J. Kim J. Cina S. Maeda Y. Barbieri R. (2013). Brain correlates of phasic autonomic response to acupuncture stimulation: An event-related fMRI study. *Hum. Brain Mapp.* 34 2592–2606. 10.1002/hbm.22091 22504841 PMC3646924

[B23] Nierhaus T. Pach D. Huang W. Long X. Napadow V. Roll S. (2015). Differential cerebral response to somatosensory stimulation of an acupuncture point vs. two non-acupuncture points measured with EEG and fMRI. *Front. Hum. Neurosci.* 9:74. 10.3389/fnhum.2015.00074 25741269 PMC4327308

[B24] Pei J. Tang L. Kang C. Shi F. Liang F. Li Y. (2025). Microneedle biosensor for real-time monitoring of hydrogen sulfide release from paraventricular nucleus of hypertension rats stimulated by electroacupuncture. *Talanta* 294:128215. 10.1016/j.talanta.2025.128215 40311479

[B25] Pock A. Niemtzow S. (2021). Acupuncture and internal medicine: Perspectives from East and West. *Med. Acupunct.* 33 3–6. 10.1089/acu.2021.29164.arp 35003486 PMC8734444

[B26] Sun R. He Z. Ma P. Yin S. Yin T. Liu X. (2021). The participation of basolateral amygdala in the efficacy of acupuncture with deqi treating for functional dyspepsia. *Brain Imaging Behav.* 15 216–230. 10.1007/s11682-019-00249-7 32125619

[B27] Wang Z. Wang X. Liu J. Chen J. Liu X. Nie G. (2017). Acupuncture treatment modulates the corticostriatal reward circuitry in major depressive disorder. *J. Psychiatr. Res.* 84 18–26. 10.1016/j.jpsychires.2016.09.014 27693978 PMC5125902

[B28] Wen Q. Ma P. Dong X. Sun R. Lan L. Yin T. (2021). Neuroimaging studies of acupuncture on low back pain: A systematic review. *Front. Neurosci.* 15:730322. 10.3389/fnins.2021.730322 34616275 PMC8488100

[B29] Wu J. Qin J. Qin J. Cheng C. Sun Z. Qin X. (2024). [Research progress on analgesic mechanism of acupuncture for cancer pain]. *Zhen Ci Yan Jiu.* 49 1220–1225. Chinese. 10.13702/j.1000-0607.20230591 39557440

[B30] Xu A. Tang Y. Zeng Q. Wang X. Tian H. Zhou Y. (2020a). Electroacupuncture enhances cognition by promoting brain glucose metabolism and inhibiting inflammation in the APP/PS1 mouse model of Alzheimer’s disease: A pilot study. *J. Alzheimers Dis.* 77 387–400. 10.3233/JAD-200242 32741819

[B31] Xu A. Zeng Q. Tang Y. Wang X. Yuan X. Zhou Y. (2020b). Electroacupuncture protects cognition by regulating tau phosphorylation and glucose metabolism via the AKT/GSK3β signaling pathway in Alzheimer’s disease model mice. *Front. Neurosci.* 14:585476. 10.3389/fnins.2020.585476 33328854 PMC7714768

[B32] Yu H. Li F. Liu J. Liu D. Guo H. Wang J. (2024). Evaluation of acupuncture efficacy in modulating brain activity with periodic-aperiodic EEG measurements. *IEEE Trans. Neural Syst. Rehabil. Eng.* 32 2450–2459. 10.1109/TNSRE.2024.3421648 38949930

[B33] Yu S. Xie M. Liu S. Guo X. Tian J. Wei W. (2020). Resting-state functional connectivity patterns predict acupuncture treatment response in primary dysmenorrhea. *Front. Neurosci.* 14:559191. 10.3389/fnins.2020.559191 33013312 PMC7506136

[B34] Zhang D. Ding G. Shen X. Yao W. Zhang Z. Zhang Y. (2008). Role of mast cells in acupuncture effect: A pilot study. *Explore* 4 170–177. 10.1016/j.explore.2008.02.002 18466847

[B35] Zhang R. Zhao Y. Wang S. (2025). Application and considerations of artificial intelligence and neuroimaging in the study of brain effect mechanisms of acupuncture and moxibustion. *Zhongguo Zhen Jiu.* 45 428–434. 10.13703/j.0255-2930.20241124-0001 40229151

[B36] Zhong L. Luo J. Ma X. Yan J. Tang Q. Bao X. (2025). Based on fNIRS technology: The effects of scalp acupuncture combined with itbs on cognitive function after stroke. *NeuroRehabilitation* 56 152–163. 10.1177/10538135241303348 40260724

[B37] Zhou Y. Liu B. Lei Y. Tang L. Li T. Yu S. (2022). Acupuncture needle-based transistor neuroprobe for in vivo monitoring of neurotransmitter. *Small* 18:e2204142. 10.1002/smll.202204142 36344461

