# Correction: Decoding the mechanisms of acupuncture by neuroimaging: an integrated review from networks to molecules

**DOI:** 10.3389/fnhum.2026.2006254

**Published:** 2026-10-09

**Authors:** Shiping Liu, Yan Bai, Jie Liu, Xia Chen, Peizhu Lv, Yulin Wang, Dandan Wang, Shun Wang

**Affiliations:** 1 Heilongjiang University of Traditional Chinese Medicine, Harbin, China; 2 Heilongjiang Provincial Academy of Traditional Chinese Medicine Sciences, Harbin, China; 3 The Second Affiliated Hospital of Heilongjiang University of Chinese Medicine, Harbin, China

**Keywords:** acupuncture, brain, EEG, fMRI, molecular mechanisms, multimodal integration, networks, neuroimaging

Due to an oversight at the manuscript submission stage, the grant number of the 2024 Provincial Key Research and Development Program was missing in the **Funding** section.

The correct **Funding** statement should read:

The author(s) declared that financial support was received for this work and/or its publication. This work was supported by the National Natural Science Foundation of China (grant no. 82474664) and 2024 Provincial Key Research and Development Program (grant no. 2023ZX06C16).

The original version of this article has been updated.

